# Do Audible Sounds During a Metacarpophalangeal and Metatarsophalangeal Thrust Manipulation Have an Impact on Intra-Articular Joint Space and Brainwave Activity?

**DOI:** 10.3390/healthcare13050554

**Published:** 2025-03-04

**Authors:** Rob Sillevis, Fransisco Selva-Sarzo, Valerie Weiss, Eleuterio A. Sanchez Romero

**Affiliations:** 1 Department of Rehabilitation Sciences, Florida Gulf Coast University, Fort Myers, FL 33965, USA; vweiss@fgcu.edu; 2 Department of Physiotherapy, University of Valencia, 46010 Valencia, Spain; info@franciscoselva.com; 3 Research Group in Nursing and Health Care, Puerta de Hierro Health Research Institute-Segovia de Arana (IDIPHISA), 28222 Majadahonda, Spain; elusanchezromero@gmail.com

**Keywords:** audible sound, thrust manipulation, autonomic nervous system, EEG, musculoskeletal ultrasound

## Abstract

Background: Joint manipulation is commonly used to manage musculoskeletal dysfunctions. Joint manipulation can result in audible sounds. The clinical significance and cause of manipulation sound remain unclear. This study aimed to identify intra-articular distance following a metacarpophalangeal (MCP) II and metatarsophalangeal (MTP) II joint manipulation in healthy subjects. Additionally, the cortical response pattern was measured. Methods: Twenty-five subjects completed this quasi-experimental repeated-measures study protocol. Musculoskeletal ultrasound was used to measure intra-articular joint distance, and a portable EEG device captured brainwave activity. The environment was controlled during testing. Initially, the joint distance of the MCP II and MTP II was measured followed by the recording of initial brainwaves. Following a pre-manipulation hold, a second brainwave measure was taken. After this, each subject underwent a thrust manipulation of either MCP II or MTP II, immediately followed by the next brainwave measurement. One minute later, the final and fourth measurement took place. Results: All subjects regardless of audible sound increased in joint space following both the MCP and MTP joint manipulation. The audible group had more significant EEG changes (*p* < 0.05) following the MCP manipulation but less following the MTP manipulation. Conclusions: This study supports the tribonucleation theory explaining audible joint manipulation sounds. The manipulation of the MCP II joint resulted in increased Theta wave activity, indicating a state of relaxation, which was larger in the audible group. The MTP II manipulation had decreased cortical effects regardless of the presence of a sound. Despite these findings, the clinical usefulness of audible sounds remains questionable.

## 1. Introduction

Manual therapy interventions, including joint manipulation, are used by various practitioners [1]. Joint manipulation has been shown to be effective in managing peripheral or spinal-joint-related stiffness or pain and can be described as skilled passive movements of a joint applied at varying speeds and amplitudes, including the small-amplitude high-velocity therapeutic (HVLT) movement [2,3,4,5]. Alternatively, joint manipulation can be described as separating two joint surfaces at variable speeds. This separation results in a stretch of the periarticular tissues, including the joint capsule. It is often accompanied by an audible sound, typically perceived by both the subject and the manipulator. Although still not understood well, the most accepted model explaining the audible sound is the mechanism of viscous adhesion or tribonucleation [6,7,8].

**Figure 1:**
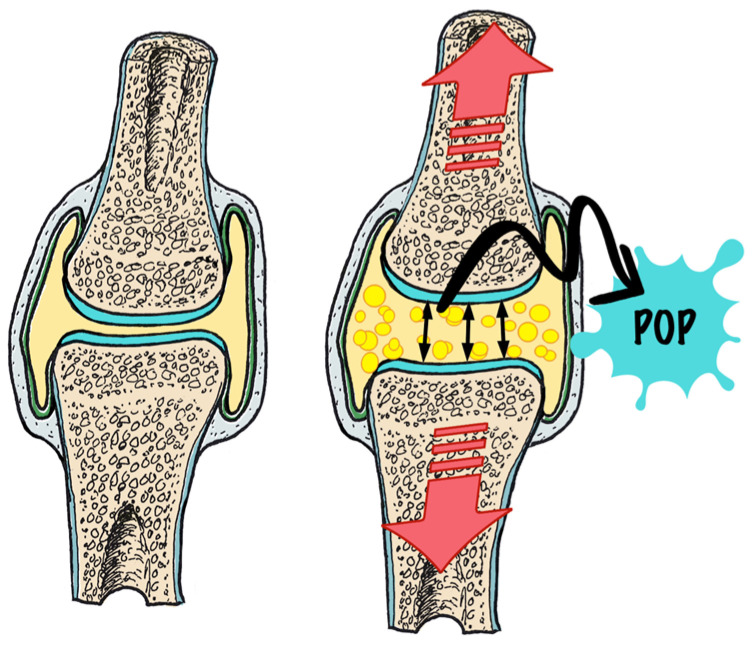
Tribonucleation. Distraction leads to development of gas bubbles and audible pop.

Tribonucleation is the process of the rapid separation of joint surfaces enclosed by the joint capsule [8,9]. If the mechanical separating force is high enough, it will overcome the intra-articular synovial tension, thus creating an overall intra-articular negative pressure. If this negative synovial pressure reaches a critical threshold, the synovial fluid will “fracture”, at which time gases already dissolved in the synovial fluid become apparent in the joint in the form of gas bubbles (Figure 1) [7].

It is believed that this synovial fluid fracture causes the commonly identified audible sound(s) often accompanying HVLT manipulation [10]. This audible sound is commonly referred to as “cavitation” [4]. The creation and presence of these intra-articular gas bubbles during and immediately after HVLT manipulation, creating an audible sound, have been demonstrated with both musculoskeletal ultrasound imaging and real-time magnetic resonance imaging [11,12]. It has been suggested that these gas bubbles consist primarily of carbon dioxide [6]. It needs to be clarified if the HVLT manipulation without audible sound also creates gas bubbles within the joint capsule. The principle of cavitation has been related to a possible destructive effect; therefore, future research should explore if HVLT manipulations are strong enough to create joint surface tissue damage [11].

**Figure 2:**
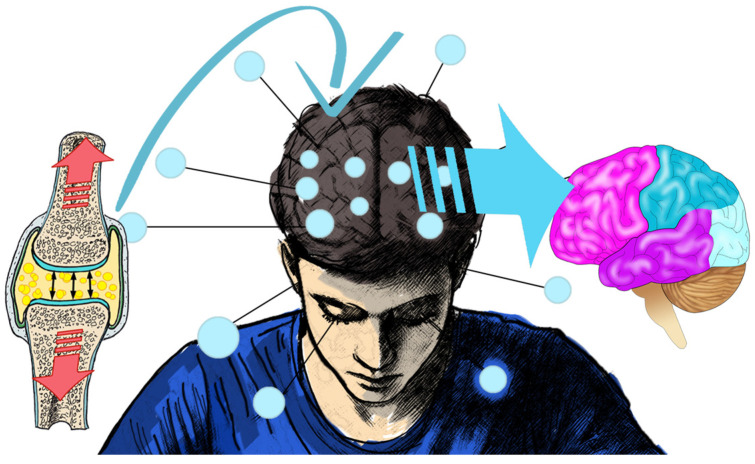
Joint manipulation leading to brain wave response and possible placebo.

Traditionally, clinicians and patients have used audible manipulation sounds as a success indicator [10,13]. Despite this claim, the true clinical relevance of the HVLT audible sound remains elusive. Every clinician has a learning curve to carry out HVLT manipulation techniques effectively [8,14]. This learning curve might contribute to not all HVLT manipulations resulting in audible sounds. Additionally, it has been reported that the audible sound only occurs in 69–70% of HVLT manipulations [13,15]. Currently, what happens inside the joint capsule following an HVLT manipulation without the production of an audible sound needs to be clarified. Neurophysiological effects have been demonstrated following HVLT manipulation without audible sounds, although this reduction in central nervous system activity appears less intense compared to the HVLT manipulation with audible sounds [16,17]. In contrast, no significant difference in autonomic nervous system activity could be identified between those without audible manipulation sound and those with a sound [13]. Neither does the audible manipulation sound correlate with improved joint range of motion, decreased pain, and reduced self-perceived disability following an HVLT manipulation [18,19]. Subjects undergoing HVLT manipulations and clinicians continue to correlate the audible pop’s presence to the manipulation’s success [9]. Therefore, the audible manipulation sound could contribute to a placebo effect (Figure 2).

However, current research efforts evaluating changes in brainwave activity do not support this hypothesis [17,20]. Bergamino et al. [9] reported that patient beliefs regarding success were directly related to the reported effectiveness of thrust manipulation. This was even though a high percentage of the patients offered rationales regarding thrust manipulation and audible sounds that were inconsistent with the current literature. If there truly is a placebo effect based on audible manipulation, sound, and changes in the central nervous system, especially cortical cortex activity post-manipulation, it should present itself. The central nervous system activity and immediate changes can be captured using electroencephalography (EEG).

Electroencephalography captures brainwave activity representing real-time neural activity changes within the subject’s cortex. Previously, EEG measurements have been used to measure cortical responses immediately following manipulative interventions [17,20]. Electrodes are placed on the scalp to measure cortical electrical conduction and potentials so that brainwave activity can be measured in each lobe. The electrodes are directly connected to an EEG processor [20]. More recently, low-cost, consumer-grade headsets have been developed. This has allowed for more practical usage in EEG research. One such device is the EPOC+ (Emotiv, San Francisco, CA, USA). The accuracy of the EEG measuring ability of the EPOC+ compared to the more traditional medical grade devices has been demonstrated [21].

The Emotiv EPOC+ consists of a 14-lead EEG unit [20]. Brainwaves are identified by their frequency and associated with the activity of specific brain regions (Table 1) [21,22]. There are five widely recognized brainwave bands: Delta, Theta, Alpha, Beta, and Gamma wave bands.

A dynamic imaging approach should be used to capture any intra-articular changes that could occur immediately following an HVLT manipulation, such as an expansion of the joint capsule. Musculoskeletal ultrasound (MSK US) imaging offers such a dynamic approach and, therefore, can be used to evaluate intra-articular HVLT manipulation responses [23,24,25,26,27,28]. Musculoskeletal ultrasound imaging is considered safe, with no known contraindications, and can be repeated easily, increasing the ability to evaluate changes in time [29]. Smith et al. [24] demonstrated the validity and reliability of MSK US imaging in viewing and measuring joint space distances directly. Ongoing technological advances have improved the visualization of both joint and capsular structures, thus increasing the accuracy of intra-articular observations and measures.

Therefore, the purpose of this study was twofold. The primary purpose was to identify intra-articular effects of an HVLT manipulation on the metacarpophalangeal (MCP) II and metatarsophalangeal (MTP) II joints for manipulations with audible sound and without in healthy asymptomatic individuals. The research hypothesis was that high-velocity thrust manipulation (HVLT) of MCP II and MTP II joints will result in intra-articular joint space changes, with or without an audible sound. A secondary purpose was to determine the cortical response pattern following an HVLT manipulation of the MCP II and MTP II joints with and without audible sounds in healthy, asymptomatic individuals. The research hypothesis was that Brainwave activity patterns will differ depending on the presence or absence of an audible cavitation sound.

## 2. Materials and Methods

### 2.1. Study Design

This quasi-experimental within-subject repeated-measures study used convenience sampling. Subjects were recruited from 1 August till 1 November 2022, from the faculty, staff, and students at Florida Gulf Coast University (Fort Myers, FL, USA). Recruitment occurred through campus-wide announcements, email communication, and flyers placed in common areas. Florida Gulf Coast University Institutional Review Board (IRB# 2020-64, date: 3 August 2021) approved this study, and all subjects provided written informed consent prior to enrollment. The study was registered with ClinicalTrials.gov (NCT04542707). In accordance with the Declaration of Helsinki, all patients signed an informed consent form before inclusion and agreed that their clinical information would be published anonymously.

#### Participants

A total of 35 potential subjects were assessed for eligibility. To be included in the study, subjects had to be between 18 and 65 years old and able to read 12th-grade English to provide proper consent. Only healthy subjects were selected for this study based on the need to minimize confounding factors such as pain-related neuroplasticity, which could influence EEG responses. Additionally, studying asymptomatic individuals provides a reference point before conducting research on pathological conditions. Exclusion criteria included recent therapeutic MCP II or MTP II joint manipulations within the last 30 days, history of surgery or fractures affecting the 2nd finger or toe, or conditions preventing HVLT manipulations. Additionally, subjects with atypical cortical neural signaling such as a history of brain injuries (e.g., concussions or traumatic brain injuries) were excluded. Of the 35 subjects assessed, 25 met the inclusion criteria and were enrolled in the study and all completed the study protocol.

### 2.2. Study Protocol

#### 2.2.1. Measurement Environment

To control for possible confounding factors effecting the EEG signal, all measurements were taken in the same research space. This allowed for the elimination of ambient noise, a controlled constant room temperature, and the room had minimal electrical interference, creating the most optimal environment for EEG measures. To avoid too much optical cortical input, the lights in the room were turned off during testing, and the subjects were only exposed to natural outside light.

#### 2.2.2. Baseline Procedures

**Figure 3:**
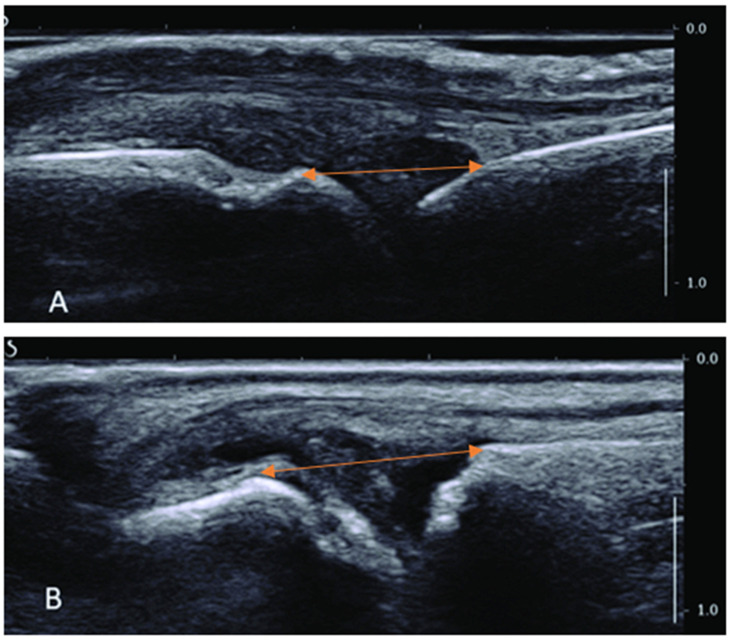
Musculoskeletal ultrasound images. Image (**A**) is dorsal side of MCP II, and Image (**B**) is dorsal side of MTP II. Orange line indicates measured joint distance.

The data collection was spread out over two testing days. Each subject entered the research room after providing consent (and ongoing consent). Each subject was instructed to assume a comfortable position and maintain this during the study protocol. While seated, the subject’s dorsal side of either the MCP II or MTP II was evaluated using a method of MSK US imaging. A direct measure of the distance between the joint partners was obtained (Figure 3). Creating reliable, repeatable images from the MCP II and MTP II joints is relatively easy. Previous studies have demonstrated that reliable images can even be obtained by novice sonographers with high intra-rater reliability (interclass coefficient greater than 0.92) [12]. Schrank et al. [30] demonstrated that reliable measures can be obtained after just 6 h of didactic training. The researcher obtaining the MSK US images during this study had significant experience using MSK US clinically since 2008 and completed an MSK US certification course.

#### 2.2.3. EEG Protocol

After the MSK US images, the subjects remained seated comfortably, at which time the EPOC+ headset was applied. Saline was used to make the EPOC+ electrodes wet enough to create optimal contact between the electrodes and the skull. The EPOC+ headset was connected to the processing Emotiv Pro software (https://www.emotiv.com) by Bluetooth. The EPOC+ concurrently measures EEG data through its 14 active electrodes with a frequency of 128 Hz. The validity of the Emotiv EPOC+ has been established previously [22,31,32]. The EPOC+-specific headset design guarantees that its 14 electrodes are placed on the head following the international standard 10–20 system for EEG measures [33]. The Emotiv EPOC+ output is provided by different wavelengths. Five different brainwaves are measured simultaneously under each electrode (Theta waves at 8–13 Hz, Alpha waves at 8–13 Hz, Beta low waves at 13–15 Hz, Beta high waves at 18–40 Hz, and Gamma waves at 40–100 Hz). The EEG measurement protocol used in this study was previously reported [17,20]. The EEG data underwent pre-filtering in the analog domain before being digitized at 128 Hz. The high-pass filter was set at 0.5 Hz, and the low-pass filter at 100 Hz to remove potential artifacts. To prevent spikes in brain activity that were not caused by the experiment from affecting the data too much, our EEG measurement protocol was time-based (15 s) for each measure. This time-based protocol should allow for more accurate EEG readings based on the true effect of the interventions. The first fifteen-second baseline EEG measure was taken (B1).

#### 2.2.4. Randomization

A randomization table was used to determine if the subject would either undergo the MCP II or MTP II manipulation on the first testing day. This randomization was applied to minimize the testing effect on the subjects.

#### 2.2.5. Intervention Procedures

On both testing days, the subject would sit comfortably with the EPOC+ in place. The subjects were instructed to not move during the measurements to eliminate movement artifacts in the EEG signal. Clinical touch from the manipulator will generate sensory input, resulting in cortical activity and thus a change in EEG data. Touch by itself has been reported to reduce stress and anxiety by possibly decreasing both Delta and Beta waves [34,35]. To minimize the effect of the newly introduced touch on brainwave activity, the manipulator would place his manipulating hand on the subject in the pre-manipulation position (not capsular end range) for three minutes. Despite this approach, it must be identified that brainwave measurements are performed in real-time; therefore, subjects could generate physiological artifacts by thought processes, which could have resulted in changes in brainwave activity and possibly result in measurement error. This could not be controlled for in this study.

After two minutes with the pre-manipulation held by the manipulator, a secondary pre-intervention fifteen-second EEG measure was taken (B2). Immediately following this measure, the subject underwent either an MCP II or the MTP II HVLT manipulation. At the start of the manipulation maneuver, the third fifteen-second EEG activity was recorded (P1). After this, the contact with the subject was maintained as a post-manipulation hold position, and the final fifteen-second EEG measure was recorded (P2). After the final measure, the EPOC+ headset was removed, and the subject’s manipulated joint was immediately re-evaluated with MSK US imaging. After this, the subject filled out a Global Rating of Change Scale (GROC) to capture their direct impression of the manipulation. Although the participants of this study were asymptomatic individuals, they could identify if something had changed following an HVLT manipulation. The GROC quantifies such change ranging from −5 to +5. Previously, the GROC was validated and shown to have high reliability with a 0.90 intraclass correlation coefficient [36]. Considering the HLVT manipulation effect was only measured immediately after the intervention and no tracking of change over time, the GROC is an appropriate tool [37,38].

#### 2.2.6. Manipulator Expertise

**Figure 4:**
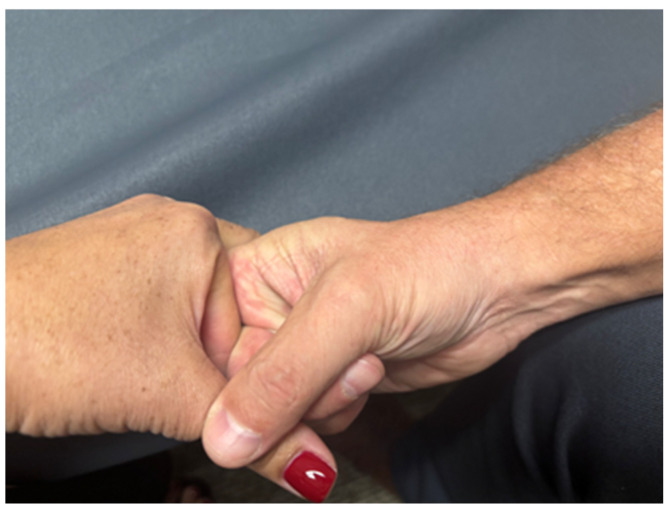
HVLT manipulation MCP II. With some traction applied through the phalanx, the operator performs a flexion-directed HVLT manipulation.

**Figure 5:**
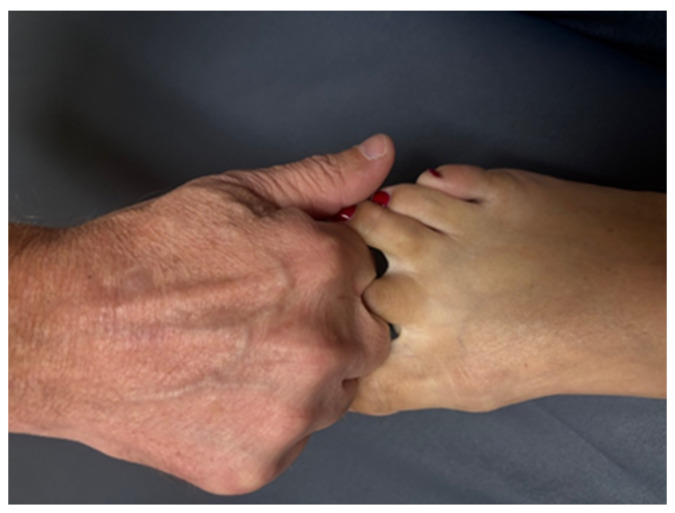
HVLT manipulation MTP II. The proximal phalanx of the second toe is placed between the proximal phalanx of the manipulator’s second and third flexed finger. A long-axis HVLT manipulation is carried out.

The researcher performing the HVLT manipulations in this study had 30 years of clinical manipulative experience and has been a fellow in the American Academy of Orthopaedic Manual Physical Therapists since 2011. By having the same manipulator perform all HVLT manipulations, variability between manipulations was minimized. The HVLT manipulation for the MCP II joint was flexion-biased, as described by Hartman [35]. During this manipulation, the researcher cradled the proximal phalanx of the second digit between his thumb and index finger (Figure 4). This hold allowed for the instruction of joint distraction followed by the HVLT manipulation in the flexion direction. The MTP II joint underwent an HVLT long-axis distraction manipulation [35]. The manipulator placed the proximal phalanx of the second toe between the proximal phalanx of his second and third flexed fingers (Figure 5). This hold allows for a good grip on the subject’s proximal phalanx. With the MTP II joint in approximately 20 degrees of flexion (caused by the manipulator hold position), an HVLT long-axis manipulation was carried out.

### 2.3. Statistical Analysis

Statistical analyses were performed using IBM^®^ SPSS^®^ Statistics version 28 with a significance level of 0.05. Descriptive statistics was used to describe the study sample. The Shapiro–Wilk test of normality identified that the MSK US imaging data were normally distributed with *p* > 0.05; for that reason, the independent sample *t*-test was used to determine the interarticular joint space difference following the manipulation and to analyze the data. The brainwaves for each of the bands (Theta, Alpha, Beta, and Gamma waves) were not normal not normally distributed with *p* < 0.05; for that reason, the Friedman’s test was used to identify any significant change between measure points. When a significance was found, the Wilcoxon’s signed rank test was used to determine where the significant difference between measures could be found. To identify if the audible manipulation sound had an effect GROC’s score, the independent sample *t*-test was used to compare the MCP II and MTP II groups.

## 3. Results

A total of 25 asymptomatic subjects completed the study protocol during a two-month period. Fourteen subjects were female (56%) and eleven were males (44%), with an age range from 22 to 33 and a mean age of 25.40 (SD = 0.54).

The mean pre-manipulation joint space distance for the MCP II audible sound group was 7.99 mm. The post-manipulation joint space distance was 9.70 mm. The mean pre-manipulation joint space distance for the MCP II non-audible sound group was 7.91 mm. The post-manipulation joint space distance was 8.06 mm. The mean pre-manipulation joint space distance for the MTP II audible sound group was 5.26 mm. The post-manipulation joint space distance was 6.46 mm. The mean pre-manipulation joint space distance for the MTP II nonaudible sound group was 5.90 mm. The post-manipulation joint space distance was 6.16 mm (Table 2).

To analyze the changes between the pre and post-manipulation joint space difference, the independent sample *t*-test was used. There was a significant difference in the pre and post-manipulation distance of MCP II in the audible group (M = 1.713, SD = 0.973); t(25) = 6.586, *p* < 0.001. There was no significant difference in pre and post-manipulation distance of MCP II in the non-audible group (M = 0.145, SD = 0.311); t(25) = 1.548, *p* = 0.153. There was a significant difference in pre and post-manipulation distance of MTP II in the audible group (M = 1.196, SD = 0.555); t(25) = 7.151 *p* < 0.001. There was a significant difference in the pre and post-manipulation distance of MTP II in the non-audible group (M = 0.265, SD = 0.273); t(25) = 3.496, *p* = 0.004 (Table 3).

**Figure 6:**
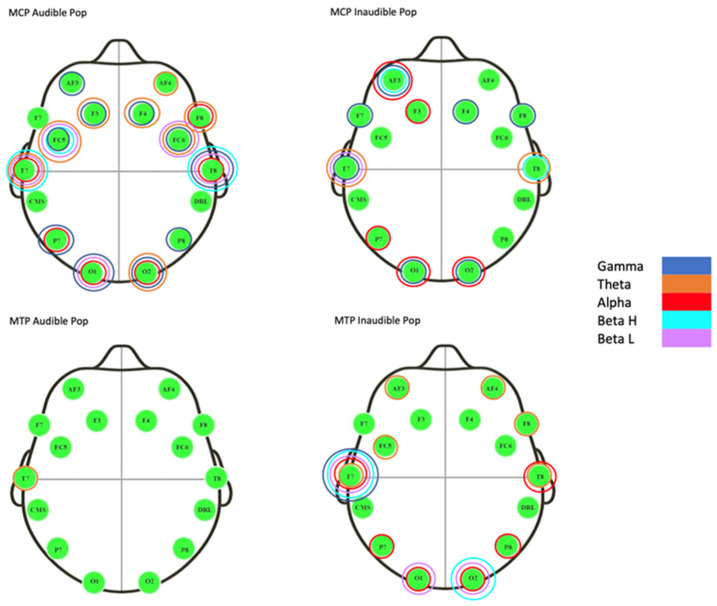
Graphical reflection of significant changes in wavebands under each electrode for both the audible and non-audible sound group. The metacarpophalangeal II audible group and the metatarsophalangeal II non-audible group displayed more significant changes in brainwave activity following the thrust manipulation.

The Theta, Alpha, Beta low, Beta high, and Gamma brainwave bands under each electrode were analyzed for normal distribution using the Shapiro–Wilk normality test. None of the wave bands were normally distributed (*p* < 0.05); therefore, the assumption for parametric statistics was not satisfied. Hence, Friedman’s test was used to determine if there was any significant change between the six measuring time points (B1-B2, B1-P1, B1-P2, B2-P1, B2-P1, and P1-P2). When significance differences were found, the Wilcoxon’s signed rank test was used to determine between what measures this significant change (*p* < 0.05) took place for each of the five wave types under each electrode (Figure 6).

Friedman’s test analyzing brainwave measurements of the HVLT manipulation of MCP II, resulting in an audible pop, showed the following electrodes and band waves to be statistically significant for change (*p* < 0.05) (Figure 6):

- Left frontal lobe: AF3 Gamma, F3 Gamma and Theta, FC5 Beta H, L, Gamma, and Theta;
- Left parietal lobe: P7 Alpha and Gamma;
- Left temporal lobe: T7 Alpha, Beta H, L, and Theta;
- Left occipital lobe: O1 Alpha, O1 Beta L, and Gamma;
- Right occipital lobe: O2 Alpha, Gamma, and Theta;
- Right temporal lobe: T8 Alpha, Beta H, and L, and Gamma;
- Right parietal lobe: P8 Gamma;
- Right frontal lobe: AF4 Theta, F4 Gamma, Theta, F8 Alpha, Theta, FC6 Beta H, Gamma, and Theta.

The Wilcoxon post-hoc analysis test was performed to identify where the significant differences were found within the Friedman test for the MCP audible group. Significant changes (*p* < 0.05) were identified between the following measure points (Table 4):

- Left frontal lobe: AF3 Gamma (B1-P1, P1-P2), F3 Gamma (B1-B2, B1-P1, P1-P2) and Theta (B2-P2), FC5 Beta H (B1, B2, B1-P1, B1-P2), L (B1, B2, B1-P1), Gamma (B1, B2, B1-P1, B2-P2), and Theta (B1, B2, B1-P1, P1-P2);
- Left parietal lobe: P7 Alpha (P1-P2) and Gamma (B1-B2, B1-P1);
- Left temporal lobe: T7 Alpha (B1-B2, B2-P1, B2-P2), Beta H (B1-B2, B2-P1, B2-P2) and L (B1-B2, B2-P1, B2-P2), and Theta (B1-B2, B2-P1, B2-P2, P1-P2);
- Left occipital lobe: O1 Alpha (B1-P1, B1-P2), O1 Beta L (B1-P2), and Gamma (B1-P1, P1-P2);
- Right occipital lobe: O2 Alpha (B1-P1, B1-P2), Gamma (B1-P1, P1-P2), and Theta (B1-P1, B1-P2, P1-P2);
- Right temporal lobe: T8 Alpha (B1-P1, B1-P2, B2-P1, B2-P2), Beta H (B1-P1, B1-P2, B2-P1) and L (B1-P1, B1-P2, B2-P1, B2-P2), and Gamma (B1-P1, B1-P2, B2-P1, B2-P2);
- Right parietal lobe: P8 Gamma (B1-P1, B1-P2, B2-P1);
- Right frontal lobe: AF4 Theta (B1-B2, B1-P1), F4 Gamma (B1-P1, P1-P2), Theta (B1-B2, B1-P1, P1-P2), F8 Alpha (B2-P1, P1-P2), Theta (B1-P1, B1-P2), FC6 Beta H (B1-P1, B1-P2), Gamma (B1-P2), and Theta (B1-P1, P1-P2).

Friedman’s test analyzing brainwave measurements of the HVLT manipulation of MCP II, resulting in the non-audible group, showed the following electrodes and band waves to be statistically significant for change (*p* < 0.05) (Figure 6):

- Left frontal lobe: AF3 Alpha, Gamma, F7 Gamma, and F3 Alpha;
- Left parietal lobe: P7 Alpha;
- Left temporal lobe: T7 Beta L, Gamma, and Theta;
- Left occipital lobe: O1 Alpha and Gamma;
- Right occipital lobe: O2 Alpha and Gamma;
- Right temporal lobe: T8 Beta H and Theta;
- Right parietal lobe: none;
- Right frontal lobe: F4 Gamma and F8 Gamma.

The Wilcoxon post-hoc analysis test was performed to identify significant differences within the Friedman test for the MCP non-audible group. Significant changes (*p* < 0.05) were identified between the following measure points (Table 5):

- Left frontal lobe: AF3 Alpha (B1-P1, B2-P2), F7 Gamma (B2-P2), and F3 Alpha (B1-P1, B1-P2);
- Left parietal lobe: P7 Alpha (B2-P1);
- Left temporal lobe: T7 Beta L (B1-B2, B2-P1), Gamma (B2-P1, P1-P2), and Theta (B1-B2, B1-P1, B2-P2);
- Left occipital lobe: O1 Alpha (B1-B2, B1-P1, B1-P2) and Gamma (B2-P2, P1-P2);
- Right occipital lobe: O2 Alpha (B1-B2, B1-P1, B1-P2) and Gamma (P1-P2);
- Right temporal lobe: T8 Beta H (B1-P1, B1-P2) and Theta (B1-B2, B1-P1, B1-P2);
- Right parietal lobe: none;
- Right frontal lobe: F4 Gamma (B1-B2) and F8 Gamma (B2-P2, P1-P2).

Friedman’s test analyzing brainwave measurements of the HVLT manipulation of MTP II, resulting in an audible pop, showed the following electrode and band wave to be statistically significant for change (*p* < 0.05) (Figure 6).

- Left temporal lobe: T7 Theta

The Wilcoxon post-hoc analysis test was performed to identify where the significant differences were found within the Friedman test for the MTP II audible group. Significant changes (*p* < 0.05) were identified between the following measure points (Table 6).

- Left temporal lobe: T7 Theta (B1-B2, B1-P1, B2-P2, P1-P2)

Friedman’s test analyzing brainwave measurements of the HVLT manipulation of MTP II for the non-audible pop showed the following electrodes and band waves to be statistically significant for change (*p* < 0.05) (Figure 4):

- Left frontal lobe: AF3 Theta and FC5 Theta;
- Left parietal lobe: P7 Alpha;
- Left temporal lobe: T7 Alpha, Beta H, L, Gamma, and Theta;
- Left occipital lobe: O1 Alpha and Beta L;
- Right occipital lobe: O2 Alpha, Beta H, and L;
- Right temporal lobe: T8 Alpha and Theta;
- Right parietal lobe: P8 Alpha;
- Right frontal lobe: AF4 Theta and F8 Theta.

The Wilcoxon post-hoc analysis test was performed to identify significant differences within the Friedman test for the MTP II non-audible group. Significant changes (*p* < 0.05) were identified between the following measure points (Table 7):

- Left frontal lobe: AF3 Theta (P1-P2) and FC5 Theta (B2-P2, P1-P2);
- Left parietal lobe: P7 Alpha (P1-P2, P1-B2, B2-P2);
- Left temporal lobe: T7 Alpha (B1-B2, B1-P1, B2-P2, P1-P2), Beta H (B1-B2, P1-P2) and L (B1-B2, B2-P2, P1-P2), Gamma (B1-B2, B1-P1, B2-P2), and Theta (B1-B2, B1-P1, B2-P2);
- Left occipital lobe: O1 Alpha (P1-P2, P1-B1, B2-P2, P1-P2) and Beta L (P1-P2, B1-P1);
- Right occipital lobe: O2 Alpha (B1-B2, P1-B1, B2-P2, P1-P2), Beta H (B1-B2, B2-P2), and L (B1-B2, B2-P1, B2-P2);
- Right temporal lobe: T8 Alpha (B1-B2) and Theta (B1-B2, B1-P1, B1-P2);
- Right parietal lobe: P8 Alpha (B1-B2, B1-P1, B2-P2, P1-P2);
- Right frontal lobe: AF4 Theta (P1-B1, P1-P2) and F8 Theta (P1-P2).

The mean GROC score for the MCP II audible sound group was 1.0. The mean GROC score for the non-audible group was 1.2. Both groups reported experiencing change. The mean GROC score for the MTP II audible group was 0.808. The mean GROC score for the non-audible group was 0.455. Both groups report a minimal experienced change (Table 8). To analyze the difference between GROC scores for the audible and non-audible groups, the independent sample *t*-test was used. There was no significant difference between the GROC scores for the MCP II audible and non-audible group with *p* = 0.569. There was no significant difference between the GROC scores for the MTP II audible and non-audible groups with *p* = 0.479 Table 9).

## 4. Discussion

This repeated-measures quasi-experimental study was designed to examine the intra-articular effects of an HVLT manipulation on the metacarpophalangeal (MCP) II and metatarsophalangeal (MTP) II joints for HVLT manipulations with and without audible sound in healthy asymptomatic individuals. A total of 25 healthy subjects underwent an HVLT flexion manipulation to the right MCP II and an HVLT distraction manipulation of the right MTP II joint. As far as we are aware, no previous study has assessed and compared changes in joint space distance and brainwave activity in relation to the presence or absence of an audible joint manipulation sound during and after HVLT manipulation of the MCP II and the MTP II joint.

The results of this study identify that an HVLT with and without audible manipulation sounds results in an overall increased joint space distance, measured using musculoskeletal ultrasound. Musculoskeletal ultrasound imaging has been identified as a safe, reliable, and valid technique to capture and measure the MCP and MTP joint space in real time [24,29]. The joint space difference was significant for the MCP II AS group, the MTP II AS, and the NAS groups. This finding agrees with previous studies that identified increased joint space following an HVLT manipulation [39]. Additionally, it concurs with previous findings that HVLT manipulation results in an increased synovial volume and, thus, capsular extension based on musculoskeletal ultrasound imaging and real-time magnetic resonance imaging studies [11,12].

The increased joint space can be explained by the fact that in both the AS and the NAS groups, an increase in synovial volume was created. The tribonucleation and cavitation theories postulate what happens after a rapid separation of joint surfaces enclosed by the joint capsule [8,9]. Based on the fact that both the AS and the NAS groups displayed an overall increased joint space distance, it does not support the cavitation theory [7,10]. The creation of gas bubbles by itself does not account for the development of audible joint manipulation sounds. This observation correlates with the findings of Kawchuk et al. [11], who, based on real-time magnetic resonance imaging, demonstrated that audible sounds are caused by cavity formation rather than cavity collapse. Our study results are consistent with the tribonucleation theory and thus support the in vivo demonstration of a synovial expansion following an HVLT manipulation. Therefore, tribonucleation should be used as the theoretical framework to explain phenomena seen with therapeutical HVLT manipulations and self-joint cracking.

A secondary purpose was to determine the cortical response pattern following an HVLT manipulation of the MCP II and MTP II joints with and without audible sounds in healthy, asymptomatic individuals. A generalized relaxation effect of HVLT manipulation has been demonstrated by a change in Alpha band brainwave activity [17,20,22]. In our subjects, a significant (*p* < 0.05) change in Alpha waves in the AS group’s left parietal, bilateral temporal lobes, and right frontal lobe was identified immediately following an HVLT manipulation of the MCP II joint. In contrast, the NAS group only displayed significant changes (*p* < 0.05) in bilateral occipital lobes and the left frontal lobe. This would imply that HVLA manipulation with audible sounds creates larger significant relaxation effect, which concurs with previous reports [17,20]. This finding should be conisidered clinically. In contrast, the HVLT manipulation of the right MTP II joint did not significantly change (*p* > 0.05) in Alpha brainwave activity in the AS group. This could imply that the more distal the HVLT manipulation is applied, the less generalized the cortical effect. In the NAS group following the HVLT of the right MTP II joint, there was a significant change (*p* < 0.05) in the bilateral parietal lobes, left temporal lobe, and bilateral occipital lobes, which could indicate that the relaxing effect of the HVLT manipulation is not dependent on the development of an audible manipulation sound.

Since the subject perceives an HVLT manipulation of the MCP II and MTP II joints as a sensory and visual input, one would expect a change in overall Gamma wave activity [17]. In the AS group, following an HVLT manipulation of the right MCP II joint, significant differences (*p <* 0.05) in Gamma waves were identified in the left frontal lobe, left parietal lobe, bilateral occipital lobes, and right parietal lobe. In the NAS group, only significant (*p* < 0.05) changes in Gamma wave activity were identified in bilateral frontal lobes, left temporal lobe, and bilateral occipital lobes. Therefore, it appears that the HVLT resulting in audible manipulation sounds creates a greater alertness and cognitive response compared to the NAS group following the MCP II HVLT manipulation. In contrast, the HVLT manipulation of the right MTP II joint did not result in any significant change (*p* > 0.05) in Alpha brainwave activity in the AS or the NAS group. This observation supports the theory that the more distal the HVLT manipulation is applied, the less generalized the cortical effect will be generated.

Bakker and Miller [40] identified the frontal lobe as a key component in the placebo effect in symptomatic individuals. Even though our sample consisted of asymptomatic individuals, an audible manipulation sound could still result in changes in brainwave activity because of expectations similar to those seen in a placebo response. Therefore, a reduction in Gamma wave activity within the frontal lobe would be anticipated. Immediately following the MCP II HLVT manipulation, Gamma brainwave activity in the frontal lobes remained comparable between both the AS and NAS groups. Additionally, neither the AS nor the NAS group had any significant changes in Gamma wave activity immediately following the MTP II HVLT manipulation. Hence, it was concluded that the audible manipulation sound likely did not produce a change in brainwave activity as typically seen in placebo responses in symptomatic individuals [17,20].

Theta waves are associated with states of deep relaxation, as well as processes involved in memory formation and learning [22]. In the AS group, following an HVLT manipulation of the right MCP II joint, significant differences (*p <* 0.05) in Theta waves were identified in bilateral frontal lobes and the left temporal lobe. In the NAS group, only significant (*p* < 0.05) changes in Gamma wave activity were identified in the left temporal lobe. There were more significant changes in the AS group, which could indicate the perception of feeling “better” and “something good happened,” resulting in a deeper level of relaxation [35,41]. This finding correlates with the results of the GROC scores, which identified that an HVLT manipulation of the MCP II resulted in a perceived change; however, it was not significant. The HVLT manipulation of the right MTP II joint did not support this observation of a deeper relaxation effect of an audible sound. A significant change (*p* > 0.05) in Theta brainwave activity was identified in the left temporal lobe in the AS group; however, in the NAS group, significant changes were seen in the bilateral frontal lobes and the right temporal lobe. This contrasts with the GROC results, which indicate a small positive but non-significant change larger in the AS than the NAS group. The observed cortical differences between the theta waves between MCP II and MTP II cannot be explained based on the study results. One hypothesis might be that this might reflect the distinct somatosensory representations of the hand and foot in the primary sensory cortex.

Conscious activities like decision-making and judgment are related to Beta wave activity [22]. In the AS group, following an HVLT manipulation of the right MCP II joint, significant differences (*p <* 0.05) in Beta waves were identified in bilateral frontal lobes and the left temporal lobe. In the NAS group, significant (*p* < 0.05) changes in Beta wave activity were identified in bilateral temporal lobes. Beta waves in the frontal lobes are similar in the AS and the NAS groups. Beta wave activity in the frontal lobes has been related to a subjective perception of pleasant stimuli [35]. This would indicate that the subjects across both groups experienced the MCP II HVLT manipulation as pleasant. The HVLT manipulation of the right MTP II joint did not result in significant changes (*p* > 0.05) in the AS group. Significant changes in the NAS group were identified in the left temporal lobe and the right occipital lobe. This could imply that the NAS group experienced the HLVT manipulation more pleasantly.

### 4.1. Limitations

This study had several limitations. First, the relatively small sample size (N = 25) may limit the generalizability of the findings, owing to the possibility of a type II error. Additionally, the sample included 14 females and 11 males, and, while the impact of gender differences might be minimal, as Ellermeier and Westphal reported no systemic differences by gender, this imbalance could still influence the results. Participants were recruited exclusively from the FGCU academic community, which is relatively homogenous in terms of health status and age. This may affect the applicability of our findings to diverse populations. Furthermore, the convenience sampling method may introduce selection bias. Future studies should consider recruiting a more diverse pool of participants to enhance external validity. Although the environment was controlled during the measurement phase of this study, it is possible that confounding factors such as ambient noise and or participant fatigue could have affected the EEG data.

Only asymptomatic subjects were selected for this study, which limits the understanding of the effects of HVLT manipulations in individuals with painful MCP II and MTP II joint conditions. Pain directly stimulates the central nervous system, potentially affecting EEG activity and GROC scores. Follow-up studies should evaluate whether a “pain subject group” responds differently to HVLT manipulation. While the study relied on human perception for the presence of audible manipulation sounds, it could be possible that some joint manipulation sounds were not identified. Future research could incorporate sound sensors for a more objective quantification of audible manipulation sounds. Additionally, the subjects in this study underwent manipulation of both MCP II and MTP II joints. The results might differ if separate cohorts are used for finger and toe manipulation. Finally, EEG data are sensitive to noise. Although the data-collection environment was designed to minimize interference, occasional noise factors could not be fully controlled.

### 4.2. Clinical Implications and Future Reserach

Further studies should build on the theoretical framework explaining the audible sounds caused by the HVLT manipulation and determine its possible physiological and neurophysiological clinical relevance. Furthermore, ongoing investigation on brainwave responses to HVLA manipulation in both symptomatic and asymptomatic individuals could possibly determine whether a consistent response pattern can be observed and how this could lead to clinical significance.

## 5. Conclusions

Thrust joint manipulation is often used to manage joint dysfunction. Audible joint sounds are often generated during HVLT manipulation. The results in our healthy asymptomatic subjects demonstrate an increase in joint space distance, visualized with musculoskeletal ultrasound imaging, in both the right MCP II and the MTP II joints in both the audible and non-audible groups. This finding supports the tribonucleation theory in explaining audible joint manipulation sounds. The EEG findings in this study demonstrated that the HVLT manipulation of the right MCP II joint resulted in a state of relaxation, which was larger when the manipulation elicited an audible sound. It appears that the manipulation of the MTP II had a decreased cortical effect regardless of the presence of an audible manipulation sound. Changes in Beta wavelength across all subjects indicate that the HVLT manipulations were pleasant overall. Further studies should correlate audible manipulation sounds with EEG activity and clinical effectiveness. Additionally, future studies should evaluate brainwave activity following an HVLT manipulation of the MCP II and MTP II joints in a symptomatic subject population.

## Figures and Tables

**Table 1:** Brainwave bands, their frequency, and the activity related with that brainwave band.

Brainwave Bands	Frequency in HZ	Representation
Delta	0.5–4	Sleep/rarely in awake individuals
Theta	4–8	Relaxation and inward focus
Alpha	8–12	Awake, often with eyes closed
Beta	13–30	Possible anxiety or external attention
Gamma	30–100	Concentration

**Table 2:** Musculoskeletal ultrasound data of the distance between intra-articular joint surface in millimeters between pre and post-manipulation for both groups. All groups displayed an increase in joint space diameter. MCP = metacarpophalangeal and MTP = metatarsophalangeal.

		Mean in mm	N	Std. Deviation	Std. Error Mean
MCP II audible sound group	Pre-distance	7.99	14	1.70	0.45
	Post-distance	9.70	14	1.99	0.53
MCP II non-audible sound group	Pre-distance	7.91	11	1.84	0.55
	Post-distance	8.06	11	1.77	0.53
MTP II audible sound group	Pre-distance	5.26	12	1.05	0.31
	Post-distance	6.46	12	1.30	0.39
MTP II non-audible sound group	Pre-distance	5.90	13	1.74	0.48
	Post-distance	6.16	13	1.83	0.50

**Table 3:** Independent *t*-test differences of pre and post-manipulation joints’ pace distance for each manipulated joint and the audible and non-audible sound groups. All groups except the metacarpophalangeal II group had a significant change in joint space diameter. Significance *p* value indicated in bold. MCP = metacarpophalangeal, MTP = metatarsophalangeal, and std = standard.

	Mean Difference After Manipulation	Std. Deviation	Std. Error Mean	95% Confidence Interval of the Difference		t	Sig. (2-Tailed)
				Lower	Upper		
MCP II audible sound group	1.713	0.973	0.260	2.275	1.151	6.586	**<0.001**
MCP II non-audible sound group	0.145	0.311	0.094	0.355	0.064	1.548	0.153
MTP II audible sound group	1.196	0.555	0.167	1.569	0.824	7.151	**<0.001**
MTP II non-audible sound group	0.265	0.273	0.076	0.430	0.100	3.496	**0.004**

**Table 4:** The Wilcoxon post-hoc analysis test results identifying between which measure points significant differences were found within the Friedman test for the metacarpophalangeal II joint audible group. B1 = pre-test 1, B2 = pre-test 2, P1 = post-test 1, and P2 = post-test 2.

Lobe	Wave Band	B1-B2	B1-P1	B1-P2	B2-P1	B2-P2	P1-P2
Left frontal	AF3		Gamma (*p* = 0.019)				Gamma (*p* = 0.026)
	FC5	Gamma (*p* = 0.013) Beta H (*p* = 0.008) Beta L (*p* = 0.03) Theta (*p* = 0.008)	Beta H (*p* = 0.008) Beta L (*p* = 0.009) Gamma (*p* = 0.008) Theta (*p* = 0.004)	Beta H (0.035)		Gamma (*p* = 0.033)	Theta (*p* = 0.019)
	F3	Gamma (*p* = 0.048) Theta (*p* = 0.004)	Gamma (*p* = 0.013) Theta (*p* = 0.001)			Theta (*p* = 0.019)	Gamma (*p* = 0.022) Theta (*p* = 0.011)
Left parietal	P7	Gamma (*p* = 0.026)	Gamma (*p* = 0.019)				Alpha (*p* = 0.035) Gamma (*p* = 0.003)
Left temporal	T7	Alpha (*p* = 0.004) Beta L (*p* = 0.004) Beta H (*p* = 0.016) Theta (*p* = 0.001)			Alpha (*p* = 0.005) Beta L (*p* = 0.003) Beta H (*p* = 0.004) Theta (*p* = 0.001)	Alpha (*p* = 0.002) Beta L (*p* = 0.009) Beta H (*p* = 0.019) Theta (*p* = 0.001)	Theta (*p* = 0.022)
Left occipital	O1		Alpha (*p* = 0.035) Gamma (*p* = 0.009)	Alpha (*p* = 0.005) Beta L (*p* = 0.002)			Gamma (*p* = 0.004)
Right occipital	O2		Alpha (*p* = 0.048) Gamma (*p* = 0.035) Theta (*p* = 0.026)	Alpha (*p* = 0.002) Theta (*p* = 0.048)			Gamma (*p* = 0.004) Theta (*p* = 0.035)
Right temporal	T8	Beta H (*p* = 0.004)	Alpha (*p* = 0.041) Beta L (*p* = 0.041) Beta H (*p* = 0.013) Gamma (*p* = 0.026)	Alpha (*p* = 0.008) Beta L (*p* = 0.013) Beta H (*p* = 0.019) Gamma (*p* = 0.026)	Alpha (*p* = 0.019) Beta L (*p* = 0.03) Beta H (*p* = 0.041) Gamma (*p* = 0.011)	Alpha (*p* = 0.011) Beta L (*p* = 0.004) Gamma (*p* = 0.008)	
Right Parietal	P8		Gamma (*p* = 0.001)	Gamma (*p* = 0.019)	Gamma (*p* = 0.048)		Gamma (*p* = 0.035)
Right frontal	AF4	Theta (*p* = 0.008)	Theta (*p* = 0.005)				Theta (*p* = 0.008)
	F8		Theta (*p* = 0.004)	Theta (*p* = 0.008)	Alpha (*p* = 0.022)		Alpha (*p* = 0.011) Theta (*p* = 0.026)
	F4	Theta (*p* = 0.035)	Gamma (*p* = 0.026) Theta (*p* = 0.019)				Gamma (*p* = 0.004) Theta (*p* = 0.019)
	FC6		Beta H (*p* = 0.008) Theta (*p* = 0.011)	Beta H (*p* = 0.009) Gamma (*p* = 0.019)			Theta (*p* = 0.048)

**Table 5:** The Wilcoxon post-hoc analysis test results identifying between which measure points significant differences were found within the Friedman test for the metacarpophalangeal II joint non-audible group. B1 = pre-test 1, B2 = pre-test 2, P1 = post-test 1, and P2 = post-test 2.

Lobe	Wave Band	B1-B2	B1-P1	B1-P2	B2-P1	B2-P2	P1-P2
Left frontal	AF3	Alpha (*p* = 0.001)	Beta H (*p* = 0.016)			Gamma (*p* = 0.033)	Gamma (*p* = 0.021)
	F7					Gamma (*p* = 0.033)	
	F3		Alpha (*p* = 0.021)	Alpha (*p* = 0.01)			
Left parietal	P7				Alpha (*p* = 0.05)		
Left temporal	T7	Beta L (*p* = 0.01) Theta (*p* = 0.016)	Theta (*p* = 0.013)		Beta L (*p* = 0.033) Gamma (*p* = 0.033)	Theta (*p* = 0.01)	Gamma (*p* = 0.021)
Left occipital	O1	Alpha (*p* = 0.013)	Alpha (*p* = 0.016)	Alpha (*p* = 0.01)		Gamma (*p* = 0.021)	Gamma (*p* = 0.041)
Right occipital	O2	Alpha (*p* = 0.004)	Alpha (*p* = 0.013)	Alpha (*p* = 0.003)			Gamma (*p* = 0.041)
Right temporal	T8	Theta (*p* = 0.003)	Beta H (*p* = 0.013) Theta (*p* = 0.026)	Beta H (*p* = 0.013) Theta (*p* = 0.01)			
Right parietal							
Right frontal	F4	Gamma (*p* = 0.033)					
	F8					Gamma (*p* = 0.033)	Gamma (*p* = 0.041)

**Table 6:** The Wilcoxon post-hoc analysis test results identifying between which measure points significant differences were found within the Friedman test for the metatarsophalangeal II joint audible group. B1 = pre-test 1, B2 = pre-test 2, P1 = post-test 1, and P2 = post-test 2.

Lobe	Wave Band	B1-B2	B1-P1	B1-P2	B2-P1	B2-P2	P1-P2
Left temporal	T7	Theta (*p* = 0.021)	Theta (*p* = 0.033)			Theta (*p* = 0.016)	Theta (*p* = 0.041)

**Table 7:** The Wilcoxon post-hoc analysis test results identifying between which measure points significant differences were found within the Friedman test for the metatarsophalangeal II joint non-audible group. B1 = pre-test 1, B2 = pre-test 2, P1 = post-test 1, and P2 = post-test 2.

Lobe	Wave Band	B1-B2	B1-P1	B1-P2	B2-P1	B2-P2	P1-P2
Left frontal	AF3						Theta (*p* = 0.013)
	FC5					Theta (*p* = 0.003)	Theta (*p* = 0.046)
Left parietal	P7	Alpha (*p* = 0.016)		Alpha (*p* = 0.006)		Alpha (*p* = 0.09)	
Left temporal	T7	Alpha (*p* = 0.019) Beta L (*p* = 0.028) Beta H (*p* = 0.023) Gamma (*p* = 0.019) Theta (*p* = 0.016)	Alpha (*p* = 0.039) Gamma (*p* = 0.046) Theta (*p* = 0.013)			Alpha (*p* = 0.009) Beta L (*p* = 0.019) Gamma (*p* = 0.019) Theta (*p* = 0.01)	Alpha (*p* = 0.011) Beta L (*p* = 0.013) Beta H (*p* = 0.046)
Left occipital	O1	Alpha (*p* = 0.002) Beta L (*p* = 0.001)	Alpha (*p* = 0.007) Beta L (*p* = 0.011)	Alpha (*p* = 0.039)		Alpha (*p* = 0.039)	Alpha (*p* = 0.019)
Right occipital	O2	Alpha (*p* = 0.001) Beta L (*p* = 0.001) Beta H (*p* = 0.019)	Alpha (*p* = 0.011)		Beta L (*p* = 0.07)	Alpha (*p* = 0.009) Beta L (*p* = 0.019) Beta H (*p* = 0.019)	Alpha (*p* = 0.039)
Right temporal	T8	Alpha (*p* = 0.002) Theta (*p* = 0.039)	Theta (*p* = 0.031)	Theta (*p* = 0.003)			
Right parietal	P8	Alpha (*p* = 0.004)	Alpha (*p* = 0.019)			Alpha (*p* = 0.023)	Alpha (*p* = 0.033)
Right frontal	F8						Theta (*p* = 0.013)
	AF4		Theta (*p* = 0.039)				Theta (*p* = 0.011)

**Table 8:** Descriptive statistics for the Global Rating of Change for the metacarpophalangeal II and metatarsophalangeal II manipulation groups. All groups reported a small but non-significant positive change after the manipulation.

		N	Mean	Std. Deviation	Std. Error Mean
GROC MCP	Audible	14	1.00	1.359	0.363
	Non-audible	11	1.20	1.398	0.442
GROC MTP II	Audible	13	0.808	1.3156	0.3649
	Non-audible	12	0.455	1.0357	0.3123

**Table 9:** Independent *t*-test comparing the difference on the Global Rating of Change score for the metacarpophalangeal II and metatarsophalangeal II manipulation groups. No significant change was identified.

		Levene’s Test for Equality of Variances		*t*-Test for Equality of Means				Std. Error of Mean
		F	Sig.	t	df	Sig. (2-Tailed)	Mean Difference	
GROC MCP II	Equal variances assumed	0.052	0.823	−0.351	22	0.729	−0.200	0.569
	Equal variances not assumed			−0.350	19.189	0.731	−0.200	0.572
GROC MTP II	Equal variances assumed	1.727	0.202	0.720	22	0.479	0.353	0.490
	Equal variances not assumed			0.735	21.911	0.470	0.353	0.480

## Data Availability

The original contributions presented in the study are included in the article; further inquiries can be directed to the corresponding author.

## Abbreviations

The following abbreviations are used in this manuscript:

HVLT	High Velocity Therapeutic
EEG	Electroencephalogram
MSK US	Musculoskeletal Ultrasound
MCP	Metacarpal Phalangeal
MTP	Metatarsal Phalangeal
GROC	Global Rating of Change
AS	Audible Sound
